# New York County

**Published:** 1897-07

**Authors:** Robert W. Ellis

**Affiliations:** Secretary


### VETERINARY MEDICAL ASSOCIATION OF NEW YORK COUNTY.

THE regular meeting was held at its rooms in the Academy of Medicine Wednesday evening, June 2d. In the absence of the President and Vice-President, Dr. J. S. Cattanach officiated at the request of the Secretary. On roll-call the following members responded: Drs. Bretherton, C. C. Cattanach, J. S. Cattanach, Ellis, Farley, Loomes, Lamkin, MacKellar, Murphy, O'Shea, and Ryder.

Dr. O'Shea, Chairman of the Judiciary Committee, reported that, although the jury bill was defeated again this year, he was informed that it would be the first exemption bill that would be obtained in this State. The Doctor also stated that it was through the efforts of some of the members of this committee that Assembly Bill No. 25, to enable Charles McCormick, of the city and county of Albany, to practice veterinary medicine and surgery as a profession was defeated in the Senate, it having passed the Assembly despite the fact that it had been opposed by members of the profession throughout the State. Moved and seconded that the report be accepted; carried.

The remainder of the evening was given up to reports of cases by members, which became very interesting. Dr. C. C. Cattanach reported a case of lockjaw and Dr. Lamkin several cow cases, all of which were freely discussed. The subject of "castration, standing," which had been discussed at the May meeting with much interest, was revived and discussed at this meeting.

Moved and seconded that the meeting adjourn, to meet again in October; carried. ROBERT W. ELLIS, D.V.S.,
Secretary.

